# Improvement in mitral regurgitation after alcohol septal ablation in hypertrophic obstructive cardiomyopathy: a retrospective cohort study

**DOI:** 10.1186/s12872-026-06952-w

**Published:** 2026-10-31

**Authors:** Anna Damlin, Andreas Rück, Mesud Mustafic, Rebecka Jandér, Anette Rickenlund, Maria J Eriksson, Nawzad Saleh, David Marlevi

**Affiliations:** 1 Department of Molecular Medicine and Surgery, Division of Clinical Physiology, Karolinska Institutet, Stockholm, Sweden; 2 Department of Cardiology, Karolinska University Hospital, Stockholm, Sweden; 3 Unit of Cardiology, Department of Medicine Solna, Karolinska Institutet, Stockholm, Sweden; 4 Department of Cardiology, Danderyds Hospital, Stockholm, Sweden; 5 Department of Clinical Physiology, Södersjukhuset, Stockholm, Sweden; 6 Department of Clinical Physiology, Karolinska University Hospital, Stockholm, Sweden; 7 Institute for Medical Engineering and Science, Massachusetts Institute of Technology, Cambridge, MA 02139 USA

**Keywords:** Alcohol septal ablation, Echocardiography, Hypertrophic obstructive cardiomyopathy, Mitral regurgitation

## Abstract

**Background:**

The primary mechanism of mitral regurgitation (MR) in hypertrophic obstructive cardiomyopathy is systolic anterior motion (SAM) of the mitral leaflets due to septal hypertrophy narrowing the left ventricular outflow tract (LVOT), influencing leaflet movement and closure. Among patients undergoing alcohol septal ablation (ASA), the impact on- and underlying mechanism of MR remains less studied. The aim was to determine the prevalence of MR and changes in MR severity after ASA and analyze echocardiographic (ECHO) parameters associated with MR changes.

**Methods:**

All patients who underwent ASA at Karolinska University Hospital in Sweden, 2009–2021 (follow-up until 2025), were included in this retrospective cohort study. ECHO parameters were collected before ASA, at 1-year follow-up and at the latest follow-up registered. To evaluate differences in continuous values, Kruskal-Wallis test was used, and for categorical values, Fischer’s exact test was used.

**Results:**

154 patients were included (mean follow-up 4.0 ± 3.9 years). 14% (*n* = 21) had at least moderate MR at baseline, of which 90% (*n* = 19) improved to mild MR or less. The patients with improved (*n* = 19, 12%) or unchanged (*n* = 128, 83%) grade of MR showed greater reduction of LVOT peak pressure gradients (improved MR: 137.5 to 4.8 mmHg, unchanged MR: 57.0 to 25.0 mmHg) between baseline and last follow-up, compared with patients with increased MR grade (*n* = 7, 4.5%, 57.0 to 51.0 mmHg, *p* = 0.003).

**Conclusions:**

Among patients with at least moderate MR before ASA, MR severity generally improved at follow-up, potentially due to septal reduction and LVOT enlargement, which may have reduced SAM.

**Clinical trial number:**

Not applicable.

**Supplementary Information:**

The online version contains supplementary material available at https://doi.org/10.1186/s12872-026-06952-w.

## Introduction

The worldwide prevalence of hypertrophic cardiomyopathy is estimated to be 1:200 to 1:500. About 60% to 70% of the patients with hypertrophic cardiomyopathy present with systolic anterior motion (SAM) of the mitral valve (MV) and left ventricular outflow tract (LVOT) obstruction, resting or provoked (inducible), hence defined as hypertrophic obstructive cardiomyopathy (HOCM) [1–3]. The pathophysiology behind SAM in HOCM comprises a mitral leaflet-septal contact during systole, together with an abnormal systolic anterior movement of the mitral leaflets towards a hypertrophic interventricular septum, narrowing the LVOT causing LVOT obstruction and impaired mitral valve closure [4, 5]. Patients with HOCM are more prone to have structural abnormalities of the MV apparatus including elongated mitral leaflets, papillary muscle malformations, displacement and/or insertion anomalies, and anomalous chordal insertions [6–8], which may contribute to SAM.

Mitral regurgitation (MR) is a common consequence of SAM with or without intrinsic structural abnormalities of the MV apparatus in HOCM [9]. The mechanism behind MR in SAM is the anterior displacement of the anterior mitral leaflet in combination with a too short, or stiff posterior mitral leaflet, causing a loss of coaptation [10]. Surgical (septal myectomy), or percutaneous (alcohol septal ablation (ASA)) reduction of the interventricular septum aiming to reduce LVOT obstruction often improves SAM and MR in patients with HOCM [11–14]. Three studies demonstrate reduction of MR after ASA, however none of the studies had MR as the primary outcome and did not further analyze the mechanism behind MR and factors associated with MR progression or reduction after ASA [15–17]. In this study, we aim to describe the changes in MR severity after ASA, focusing on the patients with MR at baseline with improvement in MR grade during follow-up after ASA.

## Methods

### Study design and population

This retrospective study comprised a population of patients with HOCM that underwent ASA on clinical indication between June 2, 2009, and July 1, 2021, at the Karolinska University Hospital in Stockholm, Sweden, which serves as a tertiary referral center, enabling treatment for patients referred from different regions in Sweden. The study population has been previously described [18]. A multi-disciplinary HCM team, including clinical and interventional cardiologists, clinical physiologists, and thoracic surgeons discussed the cases of patients who underwent ASA. The decision to proceed with ASA was based on clinical symptoms (NYHA functional class) and significant LVOT gradient as documented by rest or exercise transthoracic Doppler echocardiography (ECHO), despite optimal medical therapies including beta-blockers, calcium antagonists, and/or disopyramide, and in agreement with patient preference. Patients accepted for ASA were considered not suitable for surgical myectomy due to the high perioperative or postoperative risk because of their late age, high comorbidity, significant renal insufficiency and/or morbid obesity, or patient preference. Patients with severe preprocedural MR grade were more likely to undergo surgical myectomy to enable concomitant mitral valve surgery.

### Data collection

Patient- as well as ASA specific data were collected from patient records. All patients underwent a pre-procedural ECHO at maximum one week prior to ASA with pre-procedural ECHO parameters collected (listed in the next paragraph) as well as age, sex, length, weight, and body surface area (BSA). All patients did at least one follow-up ECHO during the follow-up period (from ASA until July 1, 2025), which was scheduled at one year after the procedure as by clinical routine. If performed, data from an additional follow-up ECHO (the latest ECHO performed during the follow-up period) was included in the study, i.e. three ECHO examinations per patient were analyzed in total. No patients were excluded due to lack of follow-up ECHO.

### Echocardiographic parameters

The ECHO parameters were collected from the imaging system (Viewpoint, GE HealthCare, U.S.A.). The image acquisition was performed according to standard protocol including additional images focusing on HOCM specific morphology and Doppler findings (as described below) to enable comprehensive HOCM evaluation [14, 19, 20]. The images were reviewed by at least one medical doctor specialized in cardiovascular imaging before the examination was finished, to enable additional images if needed. All images were retrospectively reviewed by the first author (medical doctor specialized in cardiovascular imaging), not viewing the clinical reports from the ECHO examinations prior to the review. After the study review, the study report was compared with the clinical report, and if any differences occurred, images were reviewed with an additional cardiovascular imaging specialist to reach consensus.

The data collected from the pre-procedural ECHO were heart rate, LV end-diastolic and end-systolic diameters and volumes, LV ejection fraction, E/A, E/é (measurements used in ECHO to assess left ventricular diastolic function), left atrium end-systolic volume, LV basal septal wall thickness and posterior wall thickness [20, 21]. Also, the grade of mitral- and aortic regurgitation according to the European Association of Cardiology guidelines [22], presence of SAM and LVOT peak systolic gradients, ΔP, at rest and at Valsalva maneuver, estimated from LVOT velocity (V), using the simplified Bernoulli equation (ΔP=4V^2^) were collected. LVOT obstruction was defined as LVOT peak systolic gradient *≥* 30 mmHg [14]. The length of the MV leaflets and coaptation length were manually measured [20, 23] from the pre-procedural ECHO images. The measurements of the anterior mitral leaflet (AML) and posterior mitral leaflet (PML) length were made in the apical three-chamber view at end-diastole (*n* = 145, 94.2%). Among patients with poor visualization in apical view, the measurements were performed in parasternal long axis view (PLAX) (*n* = 9, 5.8%). At follow-up, ECHO data and information about any repeat ASA or myectomy, as well as mitral valve surgery were collected. Follow-up ECHO variables were the same as from the pre-procedural ECHOs except for LV posterior wall thickness that was not collected from the follow-up ECHOs.

### Statistical analysis

The patients were grouped depending on if they had an increase in MR grade, unchanged grade of MR, or an improvement in MR grade at the last follow-up. Upon review of ECHO images and reports, intermediate grades of MR were categorized as follows: trivial to mild MR was classified as mild MR; mild to moderate as moderate MR; moderate-severe MR as severe MR [22]. Intermediate MR grades were categorized according to the higher severity grade to provide a consistent and clinically meaningful classification. This approach was selected to avoid underestimating MR severity, and because the presence of a moderate component may have greater clinical relevance than mild MR alone. The same classification principle was applied consistently across all patients to ensure comparability of MR progression and improvement. An increase in MR severity was defined as progression from no MR or mild MR on pre-procedural ECHO to moderate or severe MR on follow-up, or from pre-procedural moderate MR to severe MR on follow-up. An improvement in MR was defined as improvement from moderate MR pre-procedure to mild or no MR on follow-up, or from severe MR pre-procedure to moderate, mild, or no MR on follow-up. Patients with either no MR at baseline and mild MR at follow-up, or mild MR at baseline and no MR at follow-up were considered as unchanged MR.

Due to relatively small numbers of patients, effect sizes were presented with median and interquartile range and binary variables were reported as absolute numbers and percentages. To evaluate differences in continuous values across the three groups, Kruskal-Wallis test was used with post-hoc pairwise comparisons using Dunn’s test. To evaluate differences in categorical values across the three groups, Fischer’s exact test was used. To evaluate differences in binary values between patients with increased MR versus the patients with unchanged or improved MR grouped, univariable exact logistic regression was used. As there were quite few cases in the increased/improved MR groups, no multivariate analyses were performed. P-values < 0.05 were considered significant. Data analysis was conducted using STATA software (version 17.0 Stata Corp., College Station, Texas, USA).

## Results

**Fig. 1:**
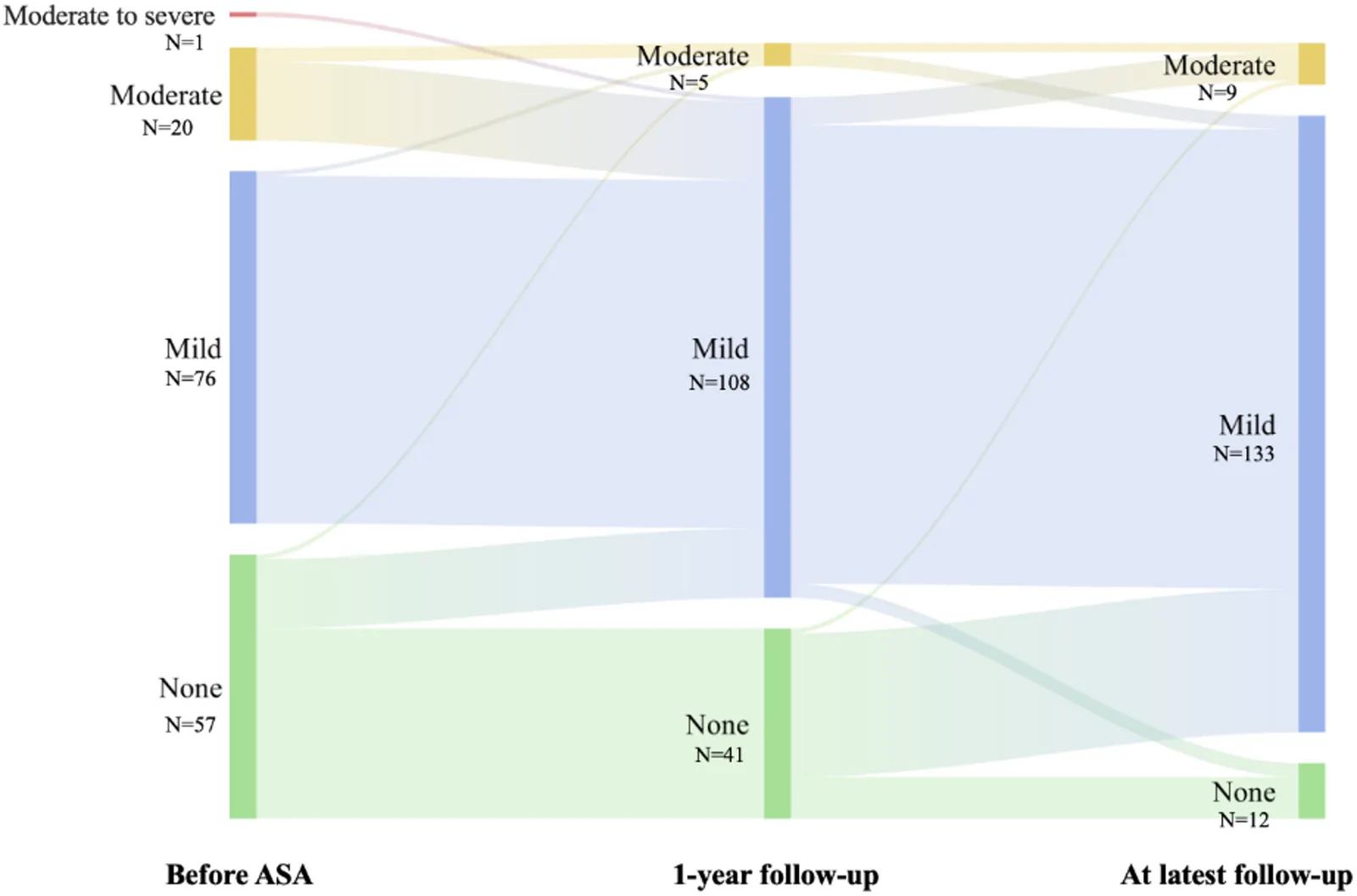
Mitral regurgitation among patients with hypertrophic obstructive cardiomyopathy prior to, and after alcohol septal ablation. Mitral regurgitation grade (none, mild, moderate, or severe MR grade) at baseline before ASA (left), 1-year follow-up (middle), and latest registered follow-up (right). Abbreviations: ASA, alcohol septal ablation; MR, mitral regurgitation

In total, 154 patients with HOCM that underwent ASA were included (Table 1). Half of the patients were women, and the median age was 63 years (interquartile range 54.0–79.0). All of the included patients had SAM, and 14% (*n* = 21) had at least moderate MR at the pre-procedural ECHO (Table 2). The mean follow-up time from ASA to the latest follow-up ECHO was 4.0 ± 3.9 years (median 2.7 years, interquartile range 1.0-5.7 years). Of the patients with at least moderate MR at the pre-procedural ECHO, 90% (*n* = 19) demonstrated improvement to mild MR or less (with no mitral valve intervention done), while 10% (*n* = 2) had unchanged moderate MR at the last follow-up (Fig. 1). At the last follow-up, 83% (*n* = 128) patients had unchanged grade of MR (no MR: 12, mild MR: 114, moderate MR: *n* = 2). In total, 5% (*n* = 7) patients increased from mild or no pre-procedural MR to moderate MR at follow-up (Table 3). No patients had severe MR at follow-up. Most patients had preprocedural beta-blocker treatment (Table 1).

**Table 1:** Clinical characteristics among patients with hypertrophic obstructive cardiomyopathy that underwent alcohol septal ablation

	All patients, *n* = 154	Increased MR after ASA, *n* = 7	Unchanged MR after ASA, *n* = 128	Improved MR after ASA, *n* = 19	*P* value
Age (years), median (IQR)	63.0 (54.0–79.0)	63.0 (53.0–69.0)	61.5 (52.0–69.0)	67.0 (57.0–74.0)	0.215
Women, n (%)	77 (50.0)	3 (42.9)	62 (48.4)	12 (63.2)	0.502
Weight (kg), median (IQR)	80.0 (70.0–95.0)	75.0 (68.0–95.0)	82.0 (71.0–96.0)	70.0 (53.0–78.0)	**0.003**
Height (cm), median (IQR)	170.0 (162.0-179.0)	174.0 (164.0-178.0)	171.0 (163.0-179.0)	167.0 (155.0-174.0)	0.243
BSA (m^2^), median (IQR)	1.9 (1.8–2.1)	1.9 (1.7–2.1)	2.0 (1.8–2.1)	1.8 (1.5-2.0)	**0.015**
Hypertension, n (%)	84 (54)	5 (71.4)	69 (53.9)	10 (52.6)	0.706
Coronary artery disease, n (%)	10 (6.5)	1 (14.3)	9 (7.0)	0 (0.0)	0.333
Atrial fibrillation*, n (%)	4 (2.6)	0 (0.0)	3 (2.3)	1 (5.3)	0.527
COPD, n (%)	1 (0.6)	0 (0.0)	1 (0.8)	0 (0.0)	1.000
**Medication at baseline**					
Beta-blocker, n (%)	145 (94.2)	7 (100.0)	120 (93.8)	18 (94.7)	1.000
Verapamil, n (%)	10 (6.5)	1 (14.3)	6 (4.7)	3 (15.8)	0.088
Amlodipine, n (%)	28 (18.2)	1 (14.3)	24 (18.8)	3 (15.8)	1.000
Diltiazem, n (%)	0 (0.0)	0 (0.0)	0 (0.0)	0 (0.0)	-
Disopyramide, n (%)	9 (5.8)	0 (0.0)	8 (6.3)	1 (5.3)	1.000

To evaluate differences in continuous values across the three groups, Kruskal-Wallis test was used. To evaluate differences in categorical values across the three groups, Fischer’s exact test was used. Significant p-values are marked in bold. *Atrial fibrillation registered during the ECHO procedure. Abbreviations: ASA, alcohol septal ablation; BSA, body surface area; COPD, chronic obstructive pulmonary disease; FU, follow-up; IQR, interquartile range; MR, mitral regurgitation

**Table 2:** Mitral regurgitation among patients with hypertrophic obstructive cardiomyopathy before alcohol septal ablation and at follow-up

	Prior to ASA, *n* (%). *N* = 154	At 1 year FU after ASA, n (%). *N* = 154	At the latest FU after ASA, n (%). *N* = 154
No regurgitation	57 (37.0)	41 (26.7)	12 (7.8)
Mild	76 (49.4)	108 (70.1)	133 (86.4)
Moderate	20 (13.0)	5 (3.2)	9 (5.8)
Moderate to severe	1 (0.6)	0 (0.0)	0 (0.0)
Severe	0 (0.0)	0 (0.0)	0 (0.0)

MR grades are ordinal. Abbreviations: ASA, alcohol septal ablation; FU, follow-up

**Table 3:** Preprocedural echocardiography parameters among patients with hypertrophic obstructive cardiomyopathy that underwent alcohol septal ablation

Pre-procedural echocardiography parameters	All patients, *n* = 154	Increased MR after ASA, *n* = 7	Unchanged MR after ASA, *n* = 128	Improved MR after ASA, *n* = 19	*P* value
LV end-diastolic volume, ml/m^2^	49.6 (37.9–58.3)	49.7 (44.9–75.7)	49.7 (36.5–58.2)	49.8 (42.5–60.0)	0.751
LV end-systolic volume, ml/m^2^	20.9 (13.9–25.5)	23.1 (12.1–35.9)	20.3 (14.2–25.0)	23.3 (12.7–26.1)	0.692
LV ejection fraction, %	60.0 (55.0–65.0)	50.0 (50.0–60.0)	60.0 (55.0–65.0)	60.0 (55.0–65.0)	0.103
LA end-systolic volume, ml/m^2^	44.5 (32.9–54.1)	43.6 (40.3–56.7)	44.3 (31.7–53.2)	53.2 (42.0-65.2)	**0.041**
BSWT, mm	19.0 (17.0–21.0)	21.0 (16.0–21.0)	19.0 (17.0-20.5)	20.0 (19.0–22.0)	0.218
LVOT gradient at rest, mmHg	29.5 (16.0–66.0)	17.0 (9.0–55.0)	25.0 (15.0–65.0)	65.0 (40.0-119.0)	**0.001**
LVOT gradient at Valsalva, mmHg	86.5 (55.5-130.5)	57.0 (22.0-100.0)	81.0 (55.0-125.0)	137.5 (85.0-182.0)	**0.023**

All continuous values are presented with median and interquartile range (IQR). To evaluate differences in continuous values across the three groups, Kruskal-Wallis test was used. Abbreviations: ASA, alcohol septal ablation; BSA, body surface area; COPD, chronic obstructive pulmonary disease; FU, follow-up; MR, mitral regurgitation

### Redo and other procedures during follow-up

Three patients underwent MV replacement with mechanical mitral prosthesis after ASA, one due to mitral valve infective endocarditis, and two due to severe obstruction and SAM despite both ASA and myectomy (all had maximum mild pre-operative MR). In total 8% (*n* = 13) patients underwent redo ASA during the follow-up period. One of these patients had increased MR at the last follow-up (despite redo ASA), one had improved MR at the last follow-up, and 6 had unchanged MR. Twelve of the patients underwent the redo ASA > 1 year after the first ASA and one patient after 0.5 years (that patient had unchanged grade of MR at both 1-year follow-up and the last follow-up). Six patients underwent surgical myectomy after ASA, 50% with no- and 50% with mild pre-procedural MR. All had unchanged MR grade at follow-up except one patient who underwent myectomy 7 months after ASA and increased from no pre-procedural MR to moderate MR 4.2 years after the myectomy. There was no difference in prevalence of comorbidities among the patients who increased in grade of MR, had unchanged grade of MR, or improved (Table 1). Symptoms at baseline and at 1-year follow-up are presented in Table S3.

### Patients with baseline moderate MR

All 21 patients that had at least moderate MR prior to ASA were evaluated as primarily SAM-mediated etiology at baseline. Degenerative abnormalities such as mitral annular calcification and leaflet degeneration were rare at baseline, whereas elongated mitral valve leaflets were more common, potential contributors to SAM (Table 4). No patient presented with pre-procedural MV prolapse, chordal rupture, leaflet destruction, or rheumatic disease, although one patient had an anomalous papillary muscle attachment, and four patients including the one with anomalous papillary muscle attachment, had exceptionally elongated chordae tendinea. At the 1-year follow-up, SAM was reduced in 86% of the 21 patients with at least moderate MR prior to ASA resulting in reduction to mild grade of MR, three had unchanged SAM and moderate MR at 1-year follow-up but reduced to mild MR at the last follow-up related to reduced SAM. At the last follow-up, two of the 18 patients who improved to mild MR at 1-year follow-up had progressed in basal septal wall thickness with worsened SAM and LVOT obstruction, resulting in moderate grade of MR again at the last follow-up (Fig. 1). That resulted in 90% of the 21 patients with at least moderate MR prior to ASA reduced to mild MR at the last follow-up.

**Table 4:** Mitral valve morphology at baseline and at follow-up after alcohol septal ablation

	All patients, *n* = 154	Increased MR after ASA, *n* = 7	Unchanged grade of MR, *n* = 128	Improved MR after ASA, *n* = 19
Baseline echocardiography				
AML (mm), median (IQR)	24.0 (22.0–26.0)	26.0 (25.0–27.0)	24.0 (22.0–26.0)	24.0 (21.0–24.0)
PML (mm), median (IQR)	16.0 (14.0–18.0)	17.5 (16.0–20.0)	16.0 (14.0–18.0)	16.0 (14.0–17.0)
PML > 13 mm, n (%)	134 (87.0)	7 (100.0)	111 (86.7)	16 (84.2)
AML > 23 mm, n (%)	95 (61.7.0)	6 (85.7)	78 (60.9)	11 (57.9)
MV coaptation length (mm), median (IQR)	9.0 (7.0–10.0)	9.0 (8.0–10.0)	9.0 (7.0–10.0)	8.0 (7.0–10.0)
Mitral annular calcification, n (%)	25 (16.2)	3 (42.9)	19 (14.8)	3 (15.8)
Degenerative mitral valve leaflet, n (%)	18 (11.7)	2 (28.6)	15 (11.7)	1 (5.3)
**Latest follow-up echocardiography**				
Mitral annular calcification, n (%)	37 (24.0)	4 (57.1)	28 (21.9)	5 (26.3)
Degenerative mitral valve leaflet, n (%)	28 (18.2)	4 (57.1)	21 (16.4)	2 (10.5)

Abbreviations: AML, anterior mitral leaflet; ASA, alcohol septal ablation; IQR, interquartile range; MR, mitral regurgitation; MV, mitral valve; PML, posterior mitral leaflet

### Comparison of improved, unchanged and increased MR groups

The follow-up time among the patients that had an increased grade of MR was 5.3 ± 4.7 years, compared with 3.1 ± 3.0 years among the patients with improved MR (*p* = 0.168), and 4.1 ± 4.0 years among those with unchanged grade of MR at the last follow-up (*p* = 0.442). When comparing the reduction in basal septal wall thickness, patients with improved MR grade tended to exhibit a larger reduction than those with increased MR grade, although the difference was not statistically significant (Table 5). The patients with improved (post-hoc Dunns test: *p* < 0.01) or unchanged (post-hoc Dunns test: *p* = 0.048) grade of MR showed larger reduction of LVOT pressure gradients at follow-up, compared with the patients with increased MR grade (Table 5). Similarly, patients with an improved MR grade demonstrated a larger reduction in LVOT gradients during the Valsalva maneuver than patients with an increased MR grade (post-hoc Dunns test: *p* = 0.02) (Table 5). The amount of alcohol delivered did not affect the outcome regarding MR (Table S1).

**Table 5:** Comparison of echocardiography parameters related to mitral regurgitation among patients undergoing alcohol septal ablation

	Increased MR after ASA, *n* = 7				Unchanged MR after ASA, *n* = 128				Improved MR after ASA, *n* = 19				
	Pre ASA	At latest FU	Δ	*P* value	Pre ASA	At latest FU	Δ	*P* value	Pre ASA	At latest FU	Δ	*P* value	*P* value*
LVEDV, ml/m^2^	49.7 (44.9–75.7)	48.0 (45.0–53.0)	5.3	0.949	49.7 (44.9–75.7)	51.6 (40.4–57.9)	0.6	**< 0.001**	49.8 (42.5–60.0)	47.4 (42.8–55.5)	0.9	0.543	0.894
LVESV, ml/m^2^	23.1 (12.1–35.9)	22.0 (19.0–22.0)	-0.8	0.848	23.1 (12.1–35.9)	19.3 (14.8–23.9)	-1.5	**< 0.001**	23.3 (12.7–26.1)	15.6 (12.3–20.2)	-3.7	**0.039**	0.565
LVEF (%)	50.0 (50.0–60.0)	55.0 (49.0–55.0)	0.0	0.948	60.0 (55.0–65.0)	60.0 (57.0–63.0)	1.0	0.351	60.0 (55.0–65.0)	60.0 (57.0–65.0)	2.0	0.539	0.931
LA ESV, ml/m	43.6 (40.3–56.7)	71.0 (56.0-76.5)	24.4	**0.048**	43.6 (40.3–56.7)	35.0 (31.0–48.0)	-4.1	0.1043	53.2 (42.0-65.2)	47.0 (41.3–54.8)	-3.1	0.485	**0.006**
BSWT, mm	21.0 (16.0–21.0)	18.0 (12.5–18.5)	-3.0	0.119	21.0 (16.0–21.0)	16.0 (13.0–18.0)	-4.0	**< 0.001**	20.0 (19.0–22.0)	14.0 (12.0–18.0)	-5.0	**< 0.001**	0.301
LVOT gradient at rest, mmHg	17.0 (9.0–55.0)	6.8 (6.3–54.3)	-4.6	0.654	17.0 (9.0–55.0)	13.0 (9.0–23.0)	-14.0	**< 0.001**	65.0 (40.0-119.0)	3.3 (2.0–10.0)	-53.6	**< 0.001**	**< 0.001**
LVOTO** at rest, n (%)	2 (28.6)	1 (14.3)	N/A	1.000	39 (30.5)	22 (17.2)	N/A	**< 0.001**	17 (89.5)	4 (21.1)	N/A	**< 0.001**	N/A
LVOT gradient at Valsalva, mmHg	57.0 (22.0-100.0)	51.0 (16.0–70.0)	-42.6	0.153	57.0 (22.0-100.0)	25.0 (15.0–70.0)	-61.0	**< 0.001**	137.5 (85.0-182.0)	4.8 (2.9–15.8)	-102.7	**< 0.001**	**0.003**
LVOTO** at Valsalva, n (%)	7 (100.0)	3 (42.9)	N/A	0.592	128 (100.0)	34 (26.6)	N/A	**< 0.001**	19 (100.0)	3 (15.8)	N/A	**< 0.001**	N/A

All continuous values are presented with median and interquartile range (IQR). Δ refers to change in continuous values per patient (follow-up – baseline), presented in median Δ. To evaluate differences in Δ change between the follow-up and baseline within each group, Kruskal-Wallis test was used for continuous values, and to evaluate differences in categorical values, Fischer’s exact test was used. *The rightmost column P-values evaluate differences in Δ change across the three groups, using Kruskal-Wallis test for continuous values, and Fischer’s exact test for categorical values. **LVOT peak systolic gradient *≥* 30 mmHg. Abbreviations: ASA, alcohol septal ablation; BL, baseline; BSA, body surface area; BSWT, basal septal wall thickness; EDV, end-diastolic volume; ESV, end-systolic volume; LA, left atrium; LV, left ventricle; LVEF, left ventricular ejection fraction; LVOT, left ventricular outflow tract; LVOTO, left ventricular outflow tract obstruction; MR, mitral regurgitation; N/A, non-applicable

### Patients with increased MR

Of the seven patients with increased MR grade, the mechanisms of MR are described in Table S2. Degenerative mitral valve disease at follow-up and elongated posterior mitral valve leaflet were more common among the patients that increased the grade of MR, compared both with patients with improved grade of MR and those with unchanged grade of MR at follow-up, respectively (Table 4). Preprocedural LV ejection fraction was lower among the patients who had increased MR at follow-up, compared with those who had improved or unchanged grade of MR at follow-up (Tables 3 and 6). When using a cutoff for enlarged LV end-diastolic (> 50 mm or > 45 mm for men and women respectively), LV ejection fraction < 55%, and elongated PML > 13 mm [21, 23], patients with increased MR grade at follow-up fulfilled these parameters to a greater extent compared with patients with improved or unchanged MR grade at follow-up (Table 6). Those who increased their MR grade after ASA had an increase in left atrial volume, compared with the unchanged and improved MR group who decreased in left atrial volume (Table 5). No significant abnormalities were seen in the aortic-, tricuspid-, or pulmonary valves.

**Table 6:** Pre-procedural echocardiography parameters among patients with increased or improved mitral regurgitation after alcohol septal ablation

	All patients, *n* = 154	Increased MR after ASA, *n* = 7	Unchanged or improved grade of MR, *n* = 147	*P*-value
Enlarged LVEDVi*	74 (48.1)	4 (57.1)	70 (47.6)	0.711
Enlarged LVEDd**	21 (13.6)	4 (57.1)	17 (11.6)	**0.007**
LVEF < 55%	27 (22.5)	4 (57.1)	23 (15.6)	**0.018**
PML > 13 mm	134 (87.0)	7 (100.0)	127 (86.4)	0.596

Values presented in numbers and percentages in parenthesis. To evaluate differences in these binary values between patients with increased MR versus the patients with unchanged or improved MR grouped, univariable exact logistic regression was used. * LVEDVi > 54 resp 45 ml/m2 for men and women, respectively. **LVEDd > 50 resp 45 mm for men and women, respectively. Abbreviations: ASA, alcohol septal ablation; LVEDd, left ventricular end-diastolic diameter; LVEDVi, left ventricular end-diastolic volume indexed for body surface area; LVEF, left ventricular ejection fraction; MR, mitral regurgitation

## Discussion

With a mean follow-up time of 4.0 ± 3.9 years after ASA, we observed an overall favorable outcome, with 90% of the patients with at least moderate MR at baseline showing an improvement in MR grade after ASA. These patients had a larger reduction of LVOT gradients, and a trend towards a greater reduction of basal septal wall thickness. However, a subset of patients (*n* = 7, 4,5%) showed the opposite pattern, developing moderate MR after ASA. These patients more frequently exhibited elongated mitral valve leaflets and degenerative valve disease, as well as less reduction of basal septal wall thickness and LVOT gradients. This suggests that septal reduction therapy was either insufficient to alleviate SAM-related MR, or that patients developed additional LV and/or MV pathology contributing to MR progression. Prior studies have reported diverging effects on MR after surgical septal myectomy: with some showing marked improvement in SAM-mediated MR, and others with residual or progressive MR over time [13, 24, 25]. To our knowledge, this study is among the few that specifically examine the outcome of MR after ASA and includes one of the largest patient cohorts to date [15–17]. Although previous studies of ASA did not designate MR as a primary outcome or assess ECHO predictors of MR progression, all studies showed reduced rates of moderate to severe MR after ASA [15–17]. A Chinese study of 46 HOCM patients undergoing ASA demonstrated a reduction from 79.1% (*n* = 34) with moderate to severe MR at baseline to 13.9% (*n* = 6) at 14 months follow-up ECHO [15]. A Portuguese study of 80 patients with mean follow-up 4.17 ± 2.13 years showed a reduction from 32.5% (*n* = 26) moderate MR at baseline to 16.3% (*n* = 13) at the 14 months follow-up ECHO [16]. A Japanese study of 44 patients with median 6 years follow-up showed a reduction from 53% (*n* = 23) moderate or severe MR at baseline to 16% (*n* = 7) at follow-up [17]. These reductions are broadly consistent with our findings, which showed a decrease from 14% (*n* = 21) with moderate or moderate-to-severe MR at baseline to 6% (*n* = 9) at last follow-up, although our cohort had a lower baseline prevalence of moderate MR.

SAM-mediated MR is the most common MR mechanism in HOCM [25]. Adequate surgical septal reduction therapy via myectomy has been shown to significantly decrease SAM-related MR [13, 24–26]. The most favorable outcomes after isolated myectomy have been observed in patients with marked septal hypertrophy, provided the procedure sufficiently enlarges the LVOT [13, 24–26]. In short, the greater the degree of septal hypertrophy, the lower the likelihood that concomitant MV intervention is required [26]. In this study, most patients with moderate MR prior to ASA demonstrated improvement in MR grade at follow-up. These patients showed reductions in basal septal wall thickness, LVOT pressure gradients, and SAM, which could possibly reflect LVOT enlargement achieved through ASA. There was a trend towards a higher proportion of NYHA class I at follow-up among patients with unchanged, or improved MR compared to patients with an increased MR grade at follow-up, however this difference did not reach statistical significance. Consistent with these findings, a higher, although not statistically significant proportion of patients in the increased MR group had persistent NYHA (New York Heart Association heart failure classification) class III symptoms at follow-up. This may be attributable to both the severity of MR grade, and persistent LVOTO in patients with increased MR grade.

Among the seven patients who developed moderate MR after ASA, three exhibited worsening SAM, three had degenerative MR (one detected at 1-year follow-up and two at final follow-up), and one developed MR secondary to left atrial enlargement. Degenerative leaflet changes and elongated posterior mitral leaflets at baseline were more prevalent among patients who increased in MR grade, compared with those with improved or unchanged grade of MR at follow-up. Three patients underwent MV replacement with a mechanical prosthesis after ASA: one due to infective endocarditis and two due to unchanged severe LVOT obstruction and SAM despite both ASA and subsequent myectomy. Notably, all three had no or only mild MR prior to ASA and at follow-up. Among the patients who increased in MR grade after ASA, several echocardiographic factors beyond basal septal wall thickness may have contributed to the worsening of MR. Lower LV ejection fraction, longer posterior mitral leaflet length, and higher prevalence of degenerative mitral valve leaflets were observed in this subgroup. Enlarged left atrial volume and reduced systolic LV function may predispose to functional MR, whereas elongated posterior leaflets may promote MR secondary to SAM or impaired MV coaptation. Importantly, these factors would not be addressed by septal reduction therapy alone. Multiple studies highlight the need for concomitant MV interventions in patients with non-SAM-dependent MR [13, 25, 26]. Identifying intrinsic MV disease and other contributors to MR prior to septal reduction therapy in HOCM is therefore important, particularly in patients with mild septal hypertrophy who may derive greater benefit from MV repair [27, 28]. Patel et al., demonstrated that patients with basal septal thickness ≤ 1.8 cm and MV anomalies frequently required concomitant MV procedures [28].

### Limitations

One limitation of this study is the single-center design, possibly limiting the generalizability. However, because this center is a tertiary referral institution receiving patients from different regions of Sweden, the study population effectively reflects a national cohort, enhancing generalizability relative to a typical single-center study. Although this is one of the largest ASA centers in Sweden, the absolute number of events (i.e., MR of at least moderate grade) was low. This limited the statistical power of the study and precluded a robust multivariable analysis. Consequently, the reported associations are based on univariate analyses and may be susceptible to confounding by baseline clinical characteristics and other factors. The relatively small number of events also limits the precision and robustness of the observed associations, and the findings should therefore be interpreted accordingly.

All ASA referrals were evaluated by our multidisciplinary team and decisions on septal reduction therapy adhered to contemporary European guidelines [14], limiting selection bias. Potential information bias related to retrospective echocardiographic review, including MR grading, was mitigated by conducting all assessments independently of clinical reports. After all echocardiograms were reviewed, any discrepancies between study and clinical reports were resolved through consensus with at least one additional cardiovascular imaging specialist, thereby minimizing misclassification, observer, and reporting bias.

## Conclusions

In this retrospective study of HOCM patients undergoing ASA, most patients with at least moderate MR prior to ASA regressed to mild or less MR at follow-up. Possibly, LVOT enlargement and reduction of SAM following septal reduction could have contributed to the regression of MR grade in these patients. However, a small subset of patients exhibited increased grade of MR, in whom intrinsic MV disease such as degenerative, or elongated mitral leaflets, along with depressed LV function, and enlarged left atrium, may have contributed to MR progression. Although studies with larger sample sizes are needed to confirm these findings, our results suggest that specific pre-procedural echocardiographic features may help identify patients at increased risk of MR deterioration after ASA, thereby improving risk stratification and informing decisions regarding concomitant MV repair or replacement.

## Electronic supplementary material

Below is the link to the electronic supplementary material.

Supplementary Material 1

## Data Availability

The datasets used and analyzed during the current study are available from the corresponding author on reasonable request.

## Abbreviations

ASA: alcohol septal ablation
ECHO: echocardiography
HOCM: hypertrophic obstructive cardiomyopathy
LVOT: left ventricular outflow tract
MR: mitral regurgitation
MV: mitral valve
SAM: systolic anterior motion

## Abbreviations

ASA: alcohol septal ablation
ECHO: echocardiography
HOCM: hypertrophic obstructive cardiomyopathy
LVOT: left ventricular outflow tract
MR: mitral regurgitation
MV: mitral valve
SAM: systolic anterior motion
