# Supplementary material for: Improvement in mitral regurgitation after alcohol septal ablation in hypertrophic obstructive cardiomyopathy: a retrospective cohort study
Source: BMC Cardiovasc Disord. 2026 Oct 31;26:1006. doi: 10.1186/s12872-026-06952-w (PMC13723101; doi:10.1186/s12872-026-06952-w)
Supplement: Supplementary file 1 [file 12872_2026_6952_MOESM1_ESM.docx]

**Supplementary tables**

**Table S1. Peri-procedural alcohol delivered during alcohol septal ablation in patients with hypertrophic obstructive cardiomyopathy.**

|  | All patients, n = 154 | Increased MR after ASA, n = 7 | Unchanged grade of MR, n = 128 | Improved MR after ASA, n = 19 | P-value |
| --- | --- | --- | --- | --- | --- |
| Amount alcohol, ml | 1.6 (1.5–1.8) | 1.4 (1.1–1.5) | 1.6 (1.5–1.8) | 1.8 (1.3–1.9) | 0.107 |
| Alcohol per septum, ml/cm | 0.9 (0.8-1.0) | 0.8 (0.5–0.9) | 0.9 (0.7-1.0) | 0.9 (0.8-1.0) | 0.312 |

Abbreviations: All values are presented with median and interquartile range (IQR). To evaluate differences in continuous values across the three groups, Kruskal-Wallis test was used. Abbreviations: ASA, alcohol septal ablation; MR, mitral regurgitation.

**Table S2. Details of the patients who increased in mitral regurgitation grade.**

|  | Baseline | | 1-year follow-up | | Last follow-up | | |  |
| --- | --- | --- | --- | --- | --- | --- | --- | --- |
| Pt no | MR grade | Mechanism of MR | MR grade | Mechanism of MR | Time to follow-up | MR grade | Mechanism of MR |  |
| 1 | Mild | MAC, degenerative leaflets | Mild | MAC, degenerative leaflets | 1 year | Moderate | SAM, MAC, degenerative leaflets |  |
| 2 | No MR | MAC, degenerative leaflets | Mild | MAC, degenerative leaflets | 3,8 years | Moderate | MAC, degenerative leaflets |  |
| 3 | Mild |  | Mild |  | 7,3 years | Moderate | SAM, degenerative leaflets |  |
| 4 | Mild | MAC | Moderate | MAC, SAM | 1,5 years | Moderate | SAM, MAC |  |
| 5 | No MR |  | Mild | SAM | 14,5 years | Moderate | Enlarged atria, SAM |  |
| 6 | Mild | Degenerative leaflets | Mild | Degenerative leaflets | 5,2 years | Moderate | Degenerative leaflets |  |
| 7 | No MR |  | No MR |  | 4,8 years | Moderate | Degenerative leaflets |  |

Abbreviations: MAC, mitral annular calcification; MR, mitral regurgitation; pt, patient; SAM, systolic anterior movement.

**Table S3. Symptoms before the procedure and at 1-year-follow-up after alcohol septal ablation.**

|  | All patients, n = 154 | Increased MR after ASA, n = 7 | Unchanged MR after ASA, n = 128 | Improved MR after ASA, n = 19 | P value |
| --- | --- | --- | --- | --- | --- |
| **Before ASA** |  |  |  |  |  |
| NYHA class I, n (%) | 0 (0.0) | 0 (0.0) | 0 (0.0) | 0 (0.0) | - |
| NYHA class II, n (%) | 13 (8.4) | 0 (0.0) | 11 (8.6) | 2 (10.5) | 0.545 |
| NYHA class III, n (%) | 136 (88.3) | 6 (85.7) | 113 (88.3) | 17 (89.5) | 1.000 |
| NYHA class IV, n (%) | 5 (3.2) | 1 (14.3) | 4 (3.1) | 0 (0.0) | 1.000 |
| **AT 1-year follow-up** |  |  |  |  |  |
| NYHA class I, n (%) | 88 (57.1) | 2 (28.6) | 76 (59.4) | 10 (52.6) | 0.270 |
| NYHA class II, n (%) | 27 (17.5) | 2 (28.6) | 18 (14.1) | 7 (36.8) | **0.036** |
| NYHA class III, n (%) | 35 (22.7) | 3 (42.9) | 30 (23.4) | 2 (10.5) | 1.000 |
| NYHA class IV, n (%) | 0 (0.0) | 0 (0.0) | 0 (0.0) | 0 (0.0) | - |

To evaluate differences across the three groups, Fischer’s exact test was used. Significant p-value is marked in bold. Abbreviations: ASA, alcohol septal ablation; MR, mitral regurgitation, NYHA, New York Heart Association heart failure classification.
